# Blindness from bilateral pseudomonas endophthalmitis following bilateral simultaneous cataract surgery: *Primum non nocere*

**DOI:** 10.5935/0004-2749.20200038

**Published:** 2020

**Authors:** Eugene R. Ting, Brendon W. H. Lee, Ivy W. Jiang, Ashish Agar, Ian C. Francis

**Affiliations:** 1 Faculty of Medicine, The University of New South Wales, Sydney, Australia; 2 Department of Ophthalmology, Prince of Wales Hospital, Sydney, Australia

The authors were impressed to read the article published by Mota on bilateral Pseudomonas endoph thalmitis following bilateral simultaneous cataract surg ery (BSCS)^(1)^. The authors’ attention was drawn to this article published by Arshinoff et al.^(2)^, in which they astutely pointed out that the article by Mota^(1)^ was a reworking of an earlier publication by the same author^(3)^.

Indeed, if the reader inspects the images of the eyes provided in both articles^(1,3)^, they appear to be the same, as Arshinoff et al. indicated^(2)^. For example, the lower temporal L-shaped limbal vessel of the right eye in the 2018 article^(1)^ is identical to that of the left eye in the 2015 article^(3)^. The main difference between the two articles was the purported age of the patient. Moreover, the authors concur with the suggestion of Arshinoff et al. that reproducing the same case 3 years later may also be interpreted as sensationalism^(2)^.

The authors noted that the incidence of postoperative endophthalmitis (POE) quoted by Mota is 0.03%0.072%^(1,3)^. This has not been the historical experience of the authors, as they have previously published an endophthalmitis rate of 0.834% in New South Wales, Australia, in 2009^(4)^. Understandably, this finding was somewhat unpopular at the time. However, it occurred precisely at the time when surgeons shifted their practice from subconjunctival scleral incisions to unsutured clear corneal incisions, especially temporal clear corneal incisions.

Readers will be familiar with large studies in Europe and smaller studies out of North America, all of which were retrospective, suggesting that intraoperative, intracameral antibiosis offered a solution to this problem. However, our group has now published over 20 peer-reviewed articles which suggest that this conclusion, although popular, may be inaccurate^(4,5)^. The reason for this is that there was no indication in any of these studies regarding closure of the wounds to prevent the ingress of potential pathogens in the first few days following surgery.

In fact, in the authors’ own article on POE^(4)^, it was clearly shown that the organisms that cause POE are not present at the time or conclusion of cataract surgery. Instead, these organisms enter the eye in the hours and days following cataract surgery. In addition, entry of Gram-negative organisms (e.g., Pseudomonas or Bacillus) into the eye to cause POE is most likely attributed to suboptimal aseptic technique at the time of surgery. This mirrors the conclusion by Arshinoff et al. regarding breaches of sterility protocol^(2)^.

However, it is far more likely that these organisms would enter the eye following surgery (rather than during surgery) in elderly patients and possibly those with mildly immunosuppressed diabetes (such as in the case reported by Mota) via digital fecal-ocular transfer. In fact, one of the senior authors in this communication emphasized to their patients that “Nothing is to touch your eye except the postoperative eyedrops”.

Apart from substandard aseptic technique, the only likely other source of such Gram-negative pathogens is from the possibly infected lacrimal drainage apparatus of the patient, of which there are no details in the report^(1)^.

It is interesting that performing BSCS results in a lower reimbursement for the surgeon than performing two operations in different treatment episodes. While this approach may appear financially attractive to the patient and the healthcare system, there are clear material risks involved.

As both Arshinoff et al. and Mota have stated^(1,2)^, BSCS is a clearly contentious issue among cataract surgeons. As Australian cataract surgeons, our group’s position is in line with that of the Royal Australian and New Zealand College of Ophthalmologists, as indicated in their preferred practice patterns statement: “Where possible, suitable time after the first eye surgery should be allowed for the onset and treatment of any of the immediate postoperative complications which may occur before second eye surgery.”

Again, as cataract surgeons, the old Latin phrase from first year medical school *“Primum non nocere”* comes rapidly to mind. To produce bilateral endophthalmitis in a patient who has entrusted his/her sight to us, let alone then be compelled to remove both of their eyes, is anathema.
