# Supplementary material for: Blindness from bilateral pseudomonas endophthalmitis following bilateral simultaneous cataract surgery: Primum non nocere
Source: Arq Bras Oftalmol. 2020 Mar-Apr;83(2):168–70. doi: 10.5935/0004-2749.20200038 (PMC11826733; doi:10.5935/0004-2749.20200038)
Supplement: Related texts

# Reply: Blindness from bilateral pseudomonas endophthalmitis following bilateral simultaneous cataract surgery: *Primum non nocere*

**Authors:** Sergio Hernandez-Da Mota

**Affiliations:** 1 Ophthalmology Service, Clinica David Unidad Oftalmologica, Morella, México

Dear Editor,

We read the input of Dr. Eugene R. Ting et al. regarding the case report titled: Bilateral Pseudomonas endophthalmitis after immediately sequential bilateral cataract surgery^(1)^.

We agree that the order of the figures may have been inadvertently and unintentionally modified, referring to the right eye as the left eye and vice-versa, when preparing or submitting the article to the journal^(1)^. However, this should not divert the attention from the fact that the primary goal of this case report was to inform a case of bilateral endophthalmitis with a catastrophic outcome after immediately sequential bilateral cataract surgery (ISBCS)^(1)^. Whether figure a is b or figure b is a is of no consequence in this case report since the aftermath was equally disastrous for both eyes.

We strongly disagree and reject the statement of Dr. Eugene R. Ting et al. implying that the motivations of the author(s) in reporting this case had to do with mere sensationalism. As pointed out in the reply to Dr. Arshinoff et al.’s letter to the editor previously published in Arquivos Brasileiros de Oftalmologia^(2)^, one of the primary purposes of reporting this case was to inform the worldwide ophthalmological community, including the Spanish-speaking ophthalmologists, regarding the possibility that catastrophic events (i.e., bilateral endophthalmitis with virulent bacteria, such as Pseudomonas aeruginosa) may occur with ISBCS.

Any of the possibilities mentioned by Dr. Eugene R. Ting et al. may have caused the endophthalmitis in both eyes, including a breach in the sterility protocol or bacterial transfer via digital-fecal-ocular transfer. Nonetheless, the author(s) were cautious and did not speculate regarding the probable causes of this catastrophic event considering the legal implications.

The position of the author(s) and the Mexican Ophthalmological Society is also in line with that of the Royal Australian and New Zealand College of Ophthalmologists’: a few weeks or at least a few days must separate the cataract surgery of one eye from the other. Once the first eye has healed with no complications, it is safer to operate the second eye.

Moreover, the legislation of many countries may consider bilateral ISBCS as an unnecessary risk for the patient and may take subsequent legal actions against the surgeon following the development of bilateral endophthalmitis.

As previously described^(2)^, it is reasonable to assume that the likelihood of an event, (e.g., bilateral endophthalmitis) occurring after ISBCS despite the implementation of a rigorous sterility protocol (though markedly low) may be present. After all, the death of both parties in a married couple is more likely to occur if they travel together, and the plane crashes than if they go on separate flights and days, even though it is a sporadic event since air travel is very safe nowadays.

Lastly, we regard the remarks that the colleague who encountered this unfortunate and unwanted complication in one of his/her patients after ISBCS, “to produce bilateral endophthalmitis,” “let alone,” and that he/she is to be anathematized, by all means, as excessive, inaccurate, and inappropriate.

Sincerely,

Sergio Hernandez-Da Mota

## References

[1] Hernandez-Da Mota S (2018). Pseudomonas aeruginosa-induced bilateral endophthalmitis
after bilateral simultaneous cataract surgery: case
report. Arq Bras Oftalmol.

[2] Arshinoff SA, Claoué C, Metha C, Johanssen B. (2019). Bilateral pseudomonas endophthalmitis after immediately
sequential bilateral cataract surgery. Arq Bras Oftalmol.
